# Region Emilia Romagna: Primary Health Care Integration/Regione Emilia-Romagna: l’integrazione nel sistema di Cure Primarie

**Published:** 2012-09-04

**Authors:** Maria Basenghi

**Affiliations:** Coordinator ‘Wide Romagna’ Health Services, Representative of Region Emilia Romagna

**Keywords:** Emilia Romagna, health care integration, primary care observatory

## Abstract

The politics of Region Emilia-Romagna have been meant to improve social and health care integration through an architecture of local services coherent with this purpose, setting up a Department dedicated to Primary Care inside the Social and Health District.

The territorial basis of the Local Health Units (LHU), the resident people, the sustained public spending, the employed human resources and the provided services all delineate the organization of the LHU. The purpose is to grant strong integration among local bodies and LHUs through a governance system (planning, management and administration) that makes a distinction between commissioning and supply. The Committenza (commissioning department), which reports to the Strategic Direction and the District, directs the offer in connection with the need analysis, whereas the Primary Care Department arranges activities and provides services by means of integrated networks which ensure continuity to the care.

The main hub in the network is known as the Casa della Salute (House of Health), which works through structured practices, protocols and procedures. LHU professionals and freelancers under contract supply primary, in-home and nursing home care, plus specialist outpatient treatment.

The Casa della Salute, whose size will depend on the context (large, medium and small), are reliable reference points for citizens, who can address to them in every moment of the day.

On behalf of the Regione Emilia-Romagna, it is the Primary Care Observatory which registers the functions existing in the 42 Houses of Health and their organizational and structural characteristics. The analysis of the obtained data will increase enhance the Houses’ implementation.

## Regione Emilia-Romagna: l’integrazione nel sistema di Cure Primarie

Le politiche della Regione Emilia-Romagna hanno inteso valorizzare l’integrazione socio-sanitaria attraverso un’architettura dei servizi locali coerente con l’obiettivo, prevedendo un Dipartimento dedicato alle cure primarie nell’ambito del Distretto.

La distribuzione territoriale delle aziende, la popolazione residente, la spesa pubblica sostenuta, le risorse umane impiegate ed i sevizi assicurati vanno a delineare l’assetto organizzativo della azienda sanitaria locale. Obiettivo è garantire forte integrazione fra enti locali e aziende attraverso un sistema di governance (pianificazione, governo e gestione) che distingue committenza e produzione. La Committenza, in capo alla Direzione Strategica e al Distretto, orienta l’offerta in relazione all’analisi del bisogno, mentre il Dipartimento delle Cure Primarie organizza ed eroga attività e servizi attraverso reti integrate che assicurano la continuità della presa in carico.

Il nodo principale della rete è la Casa della Salute che opera attraverso percorsi strutturati, protocolli e procedure. Professionisti dipendenti delle aziende e liberi professionisti convenzionati erogano assistenza primaria, domiciliare, residenziale e specialistica ambulatoriale. La Casa della Salute, dimensionata in relazione al contesto (grande, media e piccola) è punto di riferimento certo per i cittadini, al quale possono rivolgersi in ogni momento della giornata. Le funzioni presenti nelle 42 Case della Salute, le caratteristiche organizzative e strutturali e di attività sono rilevate, a livello regionale, dall’Osservatorio delle Cure Primarie. L’analisi dei dati rilevati darà ulteriore impulso alla loro implementazione.

Powerpoint presentation available at http://www.integratedcare.org at congresses – San Marino – programme.

